# Awareness of the Effect of Diabetes on Oral Health among a Population in Jeddah, Saudi Arabia

**DOI:** 10.3290/j.ohpd.a44115

**Published:** 2020-04-01

**Authors:** Ahmad H. Almehmadi, Ghada Alzaid, Sarah Quqandi, Ghaidaa Almalki, Abraar Bannan, Areej AlHindi, Abdulrahman Idrees, Anas Habiballah, Khalid Al-Shareef, Turki Alhazzazi

**Affiliations:** a Assistant Professor, Department of Oral Biology, Faculty of Dentistry, King Abdulaziz University, Jeddah, Saudi Arabia. Study idea, hypothesis, questionnaire design and validation, evaluation of the results, wrote manuscript.; b Physician, Department of Family Medicine, King Abdulaziz University Hospital, Jeddah, Saudi Arabia. Helped in questionnaire design, wrote manuscript, interpreting results.; c Dentist, Faculty of Dentistry, King Abdulaziz University, Jeddah, Saudi Arabia. Helped formulate questions and determine suitability for the study, participant recruitment, data collection, contributed to the results section.; d Assistant Professor, Department of Oral Biology, Faculty of Dentistry, King Abdulaziz University, Jeddah, Saudi Arabia. Helped prepare the study, manuscript editing, and contributed to the discussion.

**Keywords:** awareness, diabetes, knowledge, oral health

## Abstract

**Purpose:**

Diabetes is an ever-growing health issue in the Kingdom of Saudi Arabia. It has several oral health implications and oral health in turn affects diabetes control. The primary objective of this research was to study the awareness of the effect of diabetes on oral health among the general population in the city of Jeddah, Saudi Arabia.

**Materials and Methods:**

A closed-ended, validated questionnaire was distributed to 506 randomly selected shopping-mall-goers. Responses were coded and entered into spreadsheet (SPSS, IBM) and frequency distribution of the responses was calculated.

**Results:**

The majority of the respondents were females (62.5%), non-diabetic (80.2%) and reported a positive family history of diabetes (87.9%). Most of them (63.4%) understood the importance of discussing one’s diabetes status with the dentist as it affected the treatment plan, and also knew (84.4%) that diabetes affects oral health in some way. A majority also correctly responded to how diabetes affects oral health (66.3%) and to the sequelae of untreated gum disease (87.2%). The majority of the respondents had not received any tips or information regarding the connection between diabetes and oral health.

**Conclusion:**

This study reported adequate knowledge of the sample with respect to diabetes-related oral health. An important finding of this study was that the majority of the study participants did not receive information leading to diabetes-related oral health awareness or knowledge from anyone, which implies that health professionals and health media do not play the requisite role in dissemination of this important aspect of public health.

The human body is a physiological and anatomical marvel consisting of a highly sophisticated and interconnected network of innumerable biological processes. As a consequence, abnormalities that may occur in one of these processes inadvertently affects others.^30^ One bidirectional association that is frequently studied and reported in the literature is that of diabetes mellitus (DM) and oral health. Currently, close to 285 million people worldwide suffer from diabetes and these figures are expected to double by 2030.^23^ DM is a genetically and clinically heterogenous group of metabolic disorders resulting from insulin deficiency, which is caused either by an autoimmune destruction of pancreatic β-cells or increased resistance of tissues to insulin.^30,33^ The American Diabetes Association has classified DM into type 1 (immune mediated, idiopathic), type 2 (insulin resistant), as well as other specific types and gestational DM.^26^ The most common oral health complications associated with DM include xerostomia, lichen planus, salivary gland dysfunction, gingival and periodontal diseases, and marked loss of alveolar bone height.^36^

Löe^26^ described periodontitis as the sixth complication of DM. Adults with poorly controlled or uncontrolled diabetes are exposed to triple the risk of developing periodontal disease than diabetes-free individuals,^27^ and periodontitis has been reported as a major cause of tooth loss in patients with diabetes.^5,20,22^ Conversely, untreated periodontal disease contributes to worsening of glycemic control through inducing a chronic inflammatory state, which may contribute to insulin resistance.^32^ Diabetic patients are also more susceptible to dental caries subsequent to periodontal disease or xerostomia, which could be attributed to decreased salivary secretion, increased candida growth, and increased microbial colonisation (mutans streptococci and lactobacilli).^21^

Studies have shown that as much as 80% of the examined diabetic patients had oral mucosal lesions.^33^ A correlation has also been established between improved oral hygiene and glycemic control. Two systematic reviews reported that nonsurgical periodontal therapy in conjunction with or without anti-microbial therapy led to a 0.4% mean reduction in HbA1c over a 3- to 4-month follow-up in relation to no treatment.^34,35^ These findings point towards an increasing significance of oral health maintenance in patients with DM. It has been well established in literature that oral health knowledge and improving the level of awareness are prerequisites for implementing proper home oral health care regimens.^25^ In contrast, incorrect knowledge or perceived best practices, e.g. using alcohol-based mouthwashes in patients with dry mouth and discontinuation of toothbrushing if gums bleed, are in fact detrimental to the oral health of a diabetic patient and may lead to worsening of oral complications.^38^ Therefore, knowledge about the predilection for oral complications in diabetic patients as well as effective management are important.

Several studies have been conducted worldwide to evaluate the oral health awareness of diabetic patients. A study on 253 diabetic adults ranging from 22 to 87 years of age in the US revealed that more than half of the participants did not have adequate diabetes-related oral health knowledge.^38^ Another study on 101 participants aged between 31 to 79 years concluded that only 33% of participants were aware of the increased risk of periodontal disease in diabetes.^2^ Mirza et al^29^ conducted a similar study on 240 diabetic patients in Lahore, Pakistan, and found a lack of knowledge about the relationship of oral health with diabetes among the participants. Another study conducted on 405 diabetic patients in Jordan revealed that approximately 48% of the participants were aware that diabetic patients have a predilection for periodontal disease.^1^

Despite these reports, there is a scarcity of studies from the Kingdom of Saudi Arabia in relation to oral health awareness among diabetic patients. Very few studies have been conducted so far in selected cities. One such study was conducted on female diabetic patients in Riyadh, which showed that a majority of the respondents lacked knowledge about the relationship between oral health and diabetes.^7^ Another study conducted in Abha demonstrated that 52.3% of the patients were unaware of the susceptibility of diabetic patients to oral complications.^18^ The most recent study was conducted in 2015 in the city of Jeddah on patients attending the diabetes clinic of King Abdul Aziz Hospital. This study showed that 46.7% of the participants were aware of their susceptibility to gum problems if their blood sugar stayed high.^8^

The present research was also conducted in the city of Jeddah and aims to study the awareness of people about the effects of DM on oral health. This study is an update from the previous study conducted in Jeddah^8^ in terms of the time it was conducted as well as sample selection, enables reporting the current picture of diabetes-related oral health knowledge of the population.

## Materials and Methods

The present study is a descriptive, observational cross-sectional study conducted on participants in the city of Jeddah, Saudi Arabia.

A custom-designed, closed-ended, validated questionnaire was prepared in the Arabic language and consisted of sixteen questions. The first two questions were of demographic nature, while the rest addressed diabetes-related oral health knowledge. The questionnaire underwent content validity testing by distributing the questionnaire to two professional colleagues (experts in related fields) and calculating the average congruency percentage (ACP). The ACP score of the two reviewers was 0.94 (94%), which showed this questionnaire to valid for application in the present study. The questionnaire was also tested and approved by the same experts for readability, clarity of wording, and layout. After validating the questionnaire, test-retest stability was tested to measure the reliability of the questionnaire. Pearson’s correlation coefficient (r) was used to measure the stability of responses from a pilot sample of 20 randomly selected dental patients attending the Dental Outpatient Department of King Abdul Aziz University. Data from the pilot study were coded, and Pearson’s correlation coefficient was calculated for the scores of the participants, yielding overall high reliability (r = 0.87).

The final study sample comprised 506 randomly selected subjects from among shopping-mall-goers at the Red Sea Mall, Jeddah, using a simple random sampling technique. Every 3^rd^ person addressed who met the inclusion criteria and agreed to be a part of this research was incorporated into the sample until the sample size limit had been achieved.

The inclusion criteria were: all individuals older than 15 years of age who had heard about diabetes; people who could read and understand Arabic. Disabled individuals and children younger than 15 years were excluded.

Respondents known to be diabetics or non-diabetics were verbally questioned if they had been tested for diabetes in the last three months; those who said they did not know had not been tested and were unaware of their diabetic status.

The question regarding diabetes status was explained to those participants who did not know about it. Controlled blood sugar was explained as a consistent blood sugar reading of less than 140 mg/dl, while readings above this threshold with accompanying symptoms of frequent infections, tingling, and numbness in the limbs etc. were assessed as uncontrolled diabetes.

Responses were coded and entered into a spreadsheet (SPSS 23.0, IBM; Armonk, NY, USA), and frequency distribution of participants’ responses was calculated.

## Results

**Fig 1:**
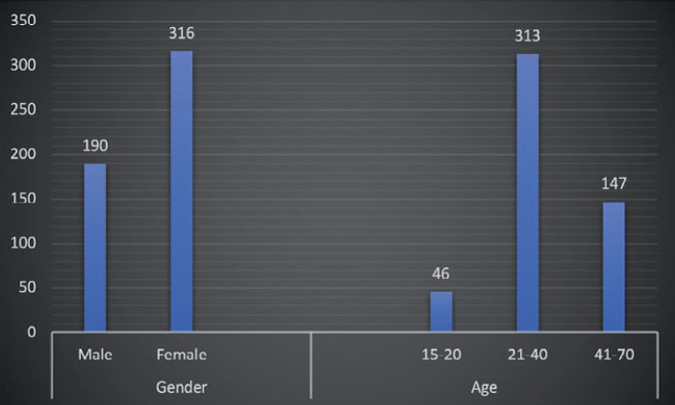
Demographics of study sample. The gender and age of respondents are shown, 62% were females and 38% were males. The respondents’ age range was from 15-70 years, 62% were 21-40 years old, 29% were 41-70 years old, and 9% were 15-20 years old.

Among the surveyed sample, the majority of the respondents, 62.5%, were female, while the rest (37.5%) were male. The demographic details are depicted in Fig 1. Most of the respondents were between 21 and 40 years of age while only 9.1% of them were 15-20 years of age. Respondents in the age group of 41-70 years accounted for 29.1% of the total sample.

**Fig 2:**
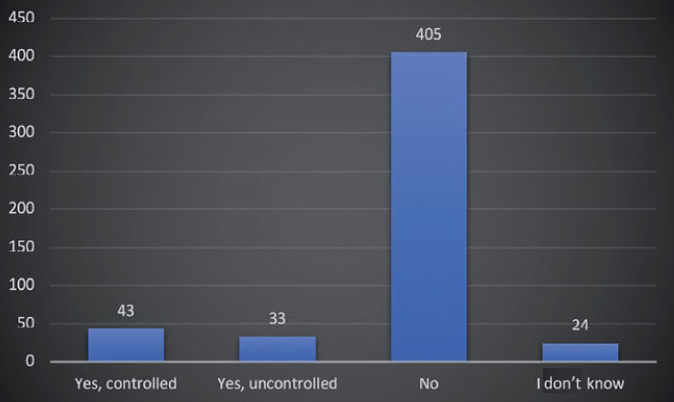
Frequency distribution of study sample by diabetes status. The participants included were diabetics, either controlled or uncontrolled, non-diabetics but with a family history of diabetes, and participants who were unaware of their diabetic condition.

**Fig 3:**
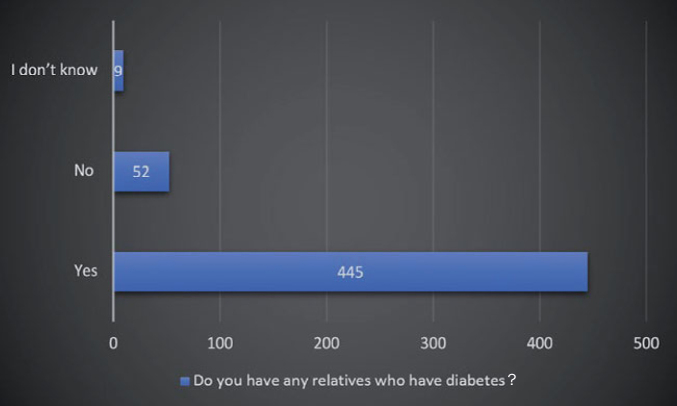
Frequency distribution of study sample by family history of diabetes. Note that the majority of participants had a family history of diabetes (88%).

Figures 2 and 3 depict the frequency distribution of the sample with respect to their diabetes status and family history of diabetes. The majority of participants in the surveyed sample were non-diabetics (80.2%). Very few of them did not know if they had diabetes or not (4.7%), and the remaining 15.1% were confirmed diabetics. Of those, 8.5% reported having controlled diabetes, while 6.6% had uncontrolled diabetes. When questioned about the family history of diabetes, 87.9% reported a positive family history, while 10.3% reported no family history of diabetes. A few (1.8%) were unaware if any of their relatives had diabetes.

The frequency distributions of participants’ responses are recorded in Table 1. Most of them (63.4%) believed that telling their dentist about their diabetic condition would affect the treatment plan, while 23.5% believed it did not. The remaining 13% did not know whether diabetes had any bearing on the treatment plan. A vast majority of the participants knew that diabetes affected the health of the mouth and teeth (84.4%), only a few did not think so (5.7%), while some of them did not know if it did or not (9.9%). 17.5% of those who responded affirmatively to the previous question did not know how diabetes affects the oral health. 66.3% of the respondents thought that diabetes reduces the body’s immune defences and delays wound healing. while the remaining 16.2% thought that diabetes attracts microbes that later cause diseases.

**Table 1:** Frequency distribution of participant responses

Question	Response	Frequency	Percentage
Gender	Male	190	37.5%
	Female	316	62.5%
Age	15-20	46	9.1%
	21-40	313	61.9%
	41-70	147	29.1%
Do you have diabetes?	Yes, controlled	43	8.5%
	Yes, uncontrolled	33	6.6%
	No	405	80.2%
	I don’t know	24	4.7%
Do you have any relatives who have diabetes?	Yes	445	87.9%
	No	52	10.3%
	I don’t know	9	1.8%
Do you think telling your dentist that you have diabetes affects your treatment plan?	Yes	321	63.4%
	No	119	23.5%
	Don’t know	66	13%
Do you think diabetes affects the health of your mouth and teeth?	Yes	427	84.4%
	No	29	5.7%
	Don’t know	50	9.9%
If you answered ‘yes’ to the previous question, then what do you think is the reason for this?	Don’t know	89	17.5%
	Reduces body’s ability to fight infection and delays wound healing	335	66.3%
	Attracts microbes that cause diseases	82	16.2%
Can uncontrolled diabetes and bad oral hygiene increase risk of having… (more than one answer possible)?	Gum bleeding, redness, swelling	217	42.9%
	Bad breath	198	39.1%
	Dry mouth	236	46.6%
	Oral infection	208	41.1%
	Tooth loss	288	56.9%
What do you think will happen if gum disease is left untreated?	Nothing	14	2.7%
	Bleeding gums will eventually stop	51	10.1%
	Gum recession, bone loss, tooth mobility	441	87.2%
If you have dry mouth, how will it affect your oral health?	It will not affect it	20	4%
	It increases risk for caries/infection	333	65.8%
	I don’t know	153	30.2%
Why do you think uncontrolled diabetics complain of dry mouth?	Side effect of drugs and elevated sugar level	308	60.9%
	Their diet	66	13%
	Inadequate drinking of water	132	26.1%
Do you think diabetes affects the healing process?	Yes	462	91.3%
	No	28	5.6%
	I don’t know	16	3.1%
Do you think diabetic patients can have dental implants?	Yes	88	17.4%
	No	59	11.7%
	I don’t know	147	29.1%
	Only if diabetes is controlled	212	41.9%
What is the most important part for having good health?	Regular check-ups with the dentist	83	16.4%
	Home care hygiene	45	8.9%
	Both	378	74.7%
If you visit the dentist regularly and maintain good oral hygiene, your blood glucose level will	Remain the same	216	42.7%
	Decrease	86	17%
	Increase	13	2.6%
	I don’t know	191	37.7%
Have you ever received tips that dental and oral health are related to diabetes?	Yes	160	31.6%
	No	346	68.4%

The responses to the risks posed by uncontrolled diabetes and poor oral hygiene were somewhat evenly distributed among participants, with tooth loss and dry mouth receiving the most responses, 56.9% and 46.6%, respectively. A small percentage (2.7%) of the participants thought that there were no sequalae of untreated gum disease, while the majority believed that it would lead to gum recession, bone loss, and tooth mobility (87.2%). The remaining 10.1% thought that bleeding would eventually stop even without treatment. Most of them believed that dry mouth increases the risk of caries and infection (65.8%), while the remaining 30.2% either did not know or did not think it had any effect (4%). The majority believed that dry mouth is either a side effect of drugs or is caused by elevated sugar levels (60.9%), while some of them (26.1%) thought that it is due to not drinking enough water. The remaining 13% believed that it is a result of the dietary habits of diabetics.

When asked about whether or not they think that diabetes affects the healing process, a vast majority (91.3%) replied in the affirmative, while 5.6% responded that they did not think so. Very few (3.1%) did not know whether it did or not. Most (41.9%) also thought that diabetics could be treated with dental implants if their blood sugar is controlled, while 11.7% thought that diabetics should not have dental implants at all. 42.7% of the participants thought that there is no impact of regular dental visits and oral hygiene maintenance on blood sugar level, while 17% believed that it would lead to a drop in blood sugar. A majority of the respondents had never received any tips or information regarding the connection between diabetes and oral health (68.4%); nonetheless, the majority (74.7%) believed that a combination of regular dental visits and a home-care regimen is the most important factor for maintaining good oral health.

## Discussion

The present study aimed to provide some insights into the awareness of a Saudi population regarding the effects of diabetes on oral health, and endeavors to make inferences for the larger population. Various studies have been conducted worldwide and in the Kingdom of Saudi Arabia on the oral health awareness of diabetic patients, but the present study went a step further and assessed the awareness of the general population, which include diabetics as well as non-diabetics. To this end, we arranged our questionnaire into four domains: a) demographic information; b) diabetes prevalence and family history; c) awareness about oral health sequalae of diabetes; and d) awareness about oral treatment in diabetes.

This study was conducted in a large shopping mall in the city of Jeddah. Mall-goers are an appropriate sample for the survey, as they are a heterogenous group of people with different socioeconomic, educational, and medical backgrounds. This heterogeneity would be difficult to achieve if, for example, the survey were conducted in a hospital or in any other public institution. The idea behind the research was to select a diverse sample that represents the population in the city of Jeddah.

In this study, 76 out of 506 (15.1%) participants were diabetic. The rest were either non-diabetic or they had no idea about their diabetes status. This is in accordance with a community-based survey conducted in Jeddah by Bahijri et al,^9^ who evaluated the glucose level of 1420 individuals and reported the diabetes prevalence to be 15.7%. However, this is in contrast to another report by Alqurashi et al,^4^ who reported a prevalence of 30% in the studied Saudi population. A systematic review conducted recently by Al-Otaibi et al^3^ reported that the nationwide prevalence rate of diabetes increased from 23.7% between 1995 and 2000 to 25.4% between 2007 and 2009. This shows the growing trend of diabetes prevalence and the need for evaluating the awareness of people regarding the relationship between DM and oral health. Even though our research shows a lower prevalence than other reports, 87.9% reported a positive family history, which implies that there is high prevalence of diabetes in the population that is not part of our research. This may present a disparity between the findings of this paper and the actual number of diabetic individuals, because research has established a strong hereditary relationship between diabetic subjects and their blood relatives. A study conducted on Moroccans suggested familial aggregation and mainly maternal transmission of type 2 diabetes in the studied population.^10^ Another study conducted on the Ethiopian population showed that diabetes was found in individuals who had diabetic first, second and third-degree relatives, indicating that heredity plays an important role in the disease.^28^ Research conducted on a very large sample of more than 150,000 diabetic subjects in Sweden found that 27.9% of the subjects had a diabetic parent or sibling, and the highest relative risk for diabetes was found in subjects with two or more diabetic siblings.^16^ Since the present research did not provide for diabetes screening of the study sample, the number of diabetic individuals cannot be conclusively established and is only based on the word of the participants. Nonetheless, the results of this paper revealed some important information pertaining to awareness among the study sample.

Dentists may need to modify the treatment plan on the basis of diabetes control and presence/absence of systemic complications.^37^ Therefore, it is imperative that patients inform their dentist if they are diabetic or not. A percentage of 63.4% of participants in this study were aware that informing the dentist about their diabetes status has an effect on the dental treatment plan. However, studies in other parts of the world suggest that patients do not talk to their dentist about their diabetes status. A study conducted in the UK revealed that 56.9% of the participants never mentioned their diabetes status to the dentist.^11^ Another study also mentioned minimal communcation about diabetes between patients and their dentists.^24^ A percentage of 84.4% of participants in the present study were aware that diabetes has a bearing on an individual’s dental health. These findings are higher than those reported in other studies, e.g. Allen et al^2^ in Ireland, who reported that only 33% of the diabetic participants were aware of the increased risk of periodontal disease in diabetes, whereas 84%, 98%, and 99% of them were aware of the increased risk of heart disease, eye diseases, and circulatory problems, respectively.^2^ Similar low awareness was reported in a study conducted on a Jordanian population by Al Habashneh et al,^1^ who reported that only 48% of the surveyed sample was aware that diabetic patients were more prone to oral diseases. Another study conducted in the UAE on 200 participants revealed that 60%, 54%, and 42% of the respondents were aware of the increased risk of periodontal disease, dental caries, and oral infections, respectively.^14^ These findings suggest that the participants in our present study were well aware of the increased risk of oral diseases in diabetes. A positive family history of diabetes in 87.9% of the participants could be the only possible reason they were aware of the correlation between diabetes and oral heath, compared to other studies which either did not study family history or did not have this high reported percentage.

The participants also displayed satisfactory knowledge as to the cause of this increased risk of oral diseases, with 66.3% of them responding that diabetes reduces the body’s ability to fight infections and delays wound healing. Salivary gland hypofunction and reduced salivary flow leading to dry mouth is one of the most common oral complications of diabetes. Patients must be made aware of the beneficial properties of saliva and the dangers of not seeking treatment for dry mouth. In our present study, when asked about what they thought were the consequences for the mouth of uncontrolled diabetes and poor oral hygiene, 46.6% of the them responded with ‘dry mouth’, while 42.9% and 56.9% of them mentioned ‘gum problems’ and ‘tooth loss’, respectively. This is important because periodontitis is the major oral complication of diabetes,^26^ and awareness regarding this complication is necessary to formulate good home oral-care strategies and achieve good glycemic control. 65.8% of the participants also thought that dry mouth increased the risk of dental caries, and 60.9% correctly answered that dry mouth is a side effect of drugs and elevated sugar levels. Other studies have reported low awareness of the implications of dry mouth on oral health.^11,14^

Imbalances in bone turnover and impaired osseous healing in relation to elevated glucose levels have been documented in many studies.^15,17^ Delayed wound healing is also a well-known characteristic of DM. In our present study, almost all participants (91.3%) replied affirmatively when asked if DM affects the healing process. This is an important finding, as an individual’s awareness of wound healing in diabetes affects their treatment choices and efforts for glycemic control. 41.9% of the participants believed that diabetic patients could be treated with dental implants if they have good glycemic control. Researchers disagree when it comes to assessing the survival rate and success of dental implants in diabetic patients, but the general belief is that dental implants survive well in patients with controlled diabetes. Several studies have reported that implant treatment in controlled diabetic patients is as successful as in non-diabeitc individuals when carried out with good metabolic control, a meticulously designed treatment plan, prophylactic measures, and adequate post-surgical maintenance.^19,31^ A systematic review conducted by Chrcanovic et al^13^ revealed that the difference between the insertion of dental implants in non-diabetic and diabetic patients did not statistically affect the implant failure rates.^13^ These studies show that implant survival is not seriously affected by the diabetes status of an individual.

Studies conducted around the world report varying sources of diabetes-related oral health information. Bahammam^8^ reported that 55.3% of the surveyed patients received diabetes-related information from family or friends while 50.9% received it from healthcare providers.^8^ Print media and television accounted for only 29.5% of awareness. In our present study, only 31.6% of participants had received tips and information about the relationship of DM and oral health. This represents an enormous information and communication gap between healthcare providers, health media, and healthcare consumers. The same has been observed by Broder et al,^12^ that health care providers do not acknowledge the interrelationship between oral health and diabetes nor do they incorporate oral health issues into diabetes screening/treatment. It is imperative that dental-care providers be mindful of specifically asking about diabetes when taking a patient’s history. Furthermore, healthcare providers need to be trained in providing information about increased risk of oral complications that can occur in diabetic patients.

A limitation of our research was that although the responses depicted awareness, no correlations could be established between the level of awareness and participant characteristics. Neither did we have an avenue for diabetic screening of individuals because the survey was not conducted in a clinical setting, and researchers did not have the resources for screening diabetics or pre-diabetic individuals in the shopping mall due to limited funding.

## Conclusion

The findings of our research indicate that there is adequate knowledge among the population regarding the association of diabetes and oral hygiene in all the domains that we studied. Our study also reported that participants did not receive information and tips about this important aspect of diabetes and oral health from external sources. It is necessary to motivate healthcare providers to impart this knowledge to their patients, and to prompt policy makers to invest in print and audio-visual media in order to engage the greater population in spreading knowledge about the diabetes-oral health connection.
